# 3D simulation of interosseous interference in sagittal split ramus osteotomy for mandibular asymmetry

**DOI:** 10.1186/s40902-023-00400-x

**Published:** 2023-09-18

**Authors:** Santhiya Iswarya Vinothini Udayakumar, Dohyoung Kim, So-Young Choi, Tae-Geon Kwon

**Affiliations:** Department of Oral and Maxillofacial Surgery, School of Dentistry, Kyungpook National University, 2177 Dalgubeol-Daero, Jung-Gu, Daegu, 41940 Republic of Korea

**Keywords:** Osteotomy, Sagittal split ramus, Asymmetry, Facial, Three-dimensional imaging, Computer assisted

## Abstract

**Background:**

The purpose of this study was to evaluate the pattern of predicted interosseous interference and to determine the influencing factor to volume of bony interference using a computer-assisted simulation system. This retrospective study recruited 116 patients with mandibular prognathism who had undergone sagittal split ramus osteotomy (SSRO) with or without maxillary osteotomy. The patients were divided into 3 groups according to the amount of menton (Me) deviation: less than 2 mm (Group 1), 2–4 mm (Group 2), and more than 4 mm (Group 3). Changes in the distal segments following BSSRO and the volume of the interosseous interference between the proximal and distal segments were simulated after matching preoperative occlusion and postoperative expected occlusion with the cone-beam computed tomography data. Ramal inclinations and other skeletal measurements were analyzed before surgery, immediately after surgery, and at least 6 months after surgery.

**Results:**

The anticipated interosseous interference was more frequently noted on the contralateral side of chin deviation (long side) than the deviated site (short side) in Groups 2 and 3. More interference volume was predicted at the long side (186 ± 343.9 mm^3^) rather than the short side (54.4 ± 124.4 mm^3^) in Group 3 (*p* = 0.033). The bilateral difference in the volume of the interosseous interference of the osteotomized mandible was significantly correlated with the Me deviation (*r* =  − 0.257, *p* = 0.009) and bilateral ramal inclination (*r* = 0.361, *p* < 0.001). The predictor variable that affected the volume of the osseous interference at each side was the amount of Me deviation (*p* = 0.010).

**Conclusion:**

By using the 3D simulation system, the potential site of bony collision could be visualized and successfully reduced intraoperatively. Since the osseous interference can be existed on any side, unilaterally or bilaterally, 3D surgical simulation is necessary before surgery to predict the osseous interference and improve the ramal inclination.

## Background

Various factors are related to successful outcomes and long-term stability after orthognathic surgery with sagittal split ramus osteotomy (SSRO). The major factor that influences the surgical outcome is the position of osteotomized segments. Inadequate position of the bone fragment including the condyle during surgery leads to postoperative problems such as malunion of the segments, temporomandibular joint (TMJ) disorders, unsatisfying skeletal contour, and inferior alveolar nerve damage.

In patients with a symmetric mandible, the mandibular rami converges anteriorly, and lesser interference occurs when the setback would be performed. In contrast, to correct the midline deviation for the patients with mandibular asymmetry, the distal segment rotates from the short side (deviated side) to the long side (contralateral side of chin deviation). This can result in premature contact of the bony segments. The amount of interference is considered important as the condyle would be dislocated from the glenoid fossa where interosseous interference is severe [1]. The proximal osteotomized segment usually rotates around the center of the condyle so that it aligns with the distal mandibular segment [2]. Displacement of proximal segment displacement caused by osseous interference may lead to early relapse and sometimes condylar torque, which can lead to TMJ disorders as a long-term complication [1, 3].

When correcting facial asymmetry, three-dimensional (3D) mandibular movement can significantly influence the mandibular angle, ramus, and chin asymmetry. According to the direction of the rotation in the axial, coronal, and sagittal planes, mandibular architecture is significantly changed by surgery. However, a simple cephalometric tracing cannot successfully predict these 3D surgical movements. These limitations can be overcome by understanding the rotational movement of the mandibular distal segment using a computer-assisted simulation system (CASS) [4–6]. The use of a CASS simplifies the process of treatment planning and virtual surgery and helps accurately reposition the osteotomized segments of the jaws [7, 8].

When using the CASS to determine the spatial orientation of the maxilla and mandible to its final position, the final occlusion is constructed first [2]. 3D evaluation of the interference between the proximal and distal segments of the mandible using a CASS preoperatively can help prevent condylar torque during surgery and influence better surgical outcome [5, 6, 9]. The CASS provides an opportunity to predict the result with increased surgical precision and low surgical risk, thus increasing the possibilities of a successful outcome [6, 10].

Prediction of interosseous interference is an important step in orthognathic surgery for patients with mandibular asymmetry. This enables the surgeon to be aware of the amount and direction of interference and to plan additional surgical procedures to eliminate interosseous interference. A few studies have mentioned the importance of evaluating the interference between the proximal and distal segments [5, 6]. Schwartz [1] suggested that rotational mandibular setback using SSRO can result in premature contact at the anterior region of a proximal segment on the long side and the posterior region on the short side. However, this conceptual remark had not been analyzed comprehensively up to now. To the best of our knowledge, the amount of interference had not been reported as volumetric data.

In the current study, we hypothesized the following: (1) there would be a difference in interosseous interference on the long side vs the short side, and (2) there would be a specific correlation between the degree of chin asymmetry and interosseous interference volume. To verify this hypothesis, this study aimed to analyze the pattern of predicted interosseous interference and evaluate the relationship between the interference volume and the Me deviation and ramal inclination using a CASS after orthognathic surgery for asymmetric mandibular prognathism. Additionally, we tested the transverse and anteroposterior stability of the chin and ramal angle to verify the feasibility of surgical reduction of osseous interference using the CASS.

## Methods

### Study subjects

This retrospective study included consecutive patients who were diagnosed with skeletal class III with or without facial asymmetry and had undergone bilateral SSRO with and without Le Fort I osteotomy at the authors’ affiliated hospital between January 2016 and December 2017. The patients with craniofacial syndrome or pathological conditions that can influence bone healing and those with postoperative follow-up records of less than 6 months were excluded. All surgical procedures were performed by one operator (T. G. K). The Institutional Review Board of Kyungpook National University Dental Hospital reviewed and approved the study design (KNUDH-2018–05-005).

Previous studies reported that over than 2–4-mm chin deviation was regarded as facial asymmetry [11, 12]. In this study, the patients were grouped according to the absolute amount of menton (Me) deviation: Group 1, patients with no asymmetry (Me deviation < 2 mm); Group 2, patients with mild asymmetry (Me deviation 2–4 mm); and Group 3, patients with significant asymmetry (Me deviation > 4 mm).

### 3D CT image reconstruction and surgical planning

Conventional cone beam computed tomography (CBCT) was performed at three-time intervals [before surgery (T0), immediately after surgery (T1), and at least 6 months after surgery (T2)] using a CB MercuRay scanner (Hitachi Medical Corporation, Tokyo, Japan). Preoperative radiographs were taken at 1 month prior to surgery. On the other hand, postoperative radiographs were taken at 6 to 12 months after surgery.

3D facial skeletal images were reconstructed with CBCT data using 3D software (SimPlant O&O, Materialise, Leuven, Belgium or OnDemand, CyberMed Inc., Seoul, Korea). Preoperative maxillomandibular dental plaster models and occlusal relationship were scanned using a 3D model scanner (Maestro 3D scan; Cep Tech, Seoul, Korea). Preoperative 3D CT data and virtual dental models were superimposed. After completion of integrated 3D dentition-skeletal complex data before surgery, virtual skeletal surgery was performed with 3D-segmented mandible and maxilla. Each skeletal segment was mobilized according to the planned postoperative occlusion. The intermediate or final occlusal splint was virtually designed using a computer and a manufacturing system (Geomagic Control X, 3D Systems Inc., Rock Hill, SC, USA). The splint was fabricated using photoactivated resin (Accura SI 40 Nd-type stereolithography resin; 3D Systems, Valencia, CA, USA).

**Fig. 1:**
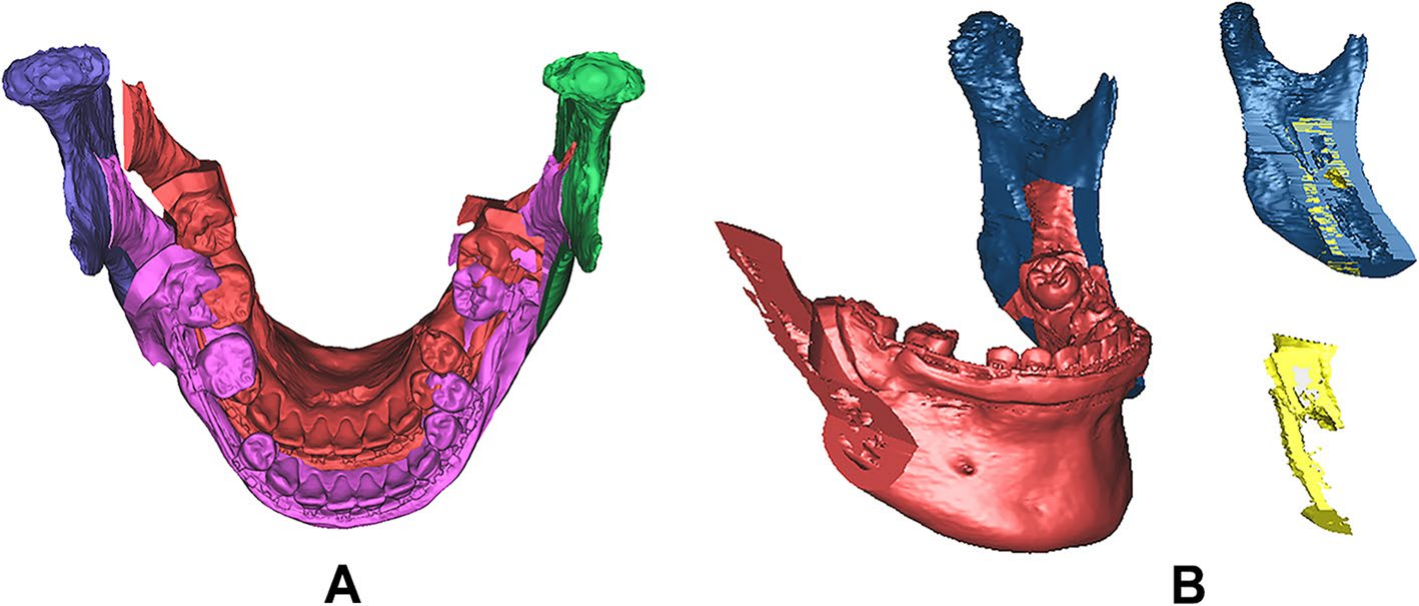
**A** Computer-assisted surgical simulation of sagittal split ramus osteotomy based on changes in presurgical and predicted postsurgical occlusion (red, preoperative distal segment; purple, predicted postsurgical distal segment). **B** Evaluation of the volume of interference. The intersection of the proximal and distal osteotomized segments to evaluate the interosseous interference. In this example, the volume of interference was 531.92 mm^3^

The planned postoperative 3D dentition-skeletal complex position was compared with the presurgical or original position (Fig. 1A). The CASS was used with 3D software (SimPlant O&O, Materialise, Leuven, Belgium, and Mimics, v19.0, Materialise, Belgium).

### Computer-assisted simulation and volumetric measurements of interosseous interference

The 3D volume of the interosseous interference was evaluated using the 3D software (Mimics, version 19.0, Materialise, Belgium). The volume was measured and analyzed according to the mobilization of the osteotomized distal segment. Then, interferences between the mobilized mandibular distal segment with mandibular dentition and the planned postoperative occlusion and mandibular proximal segments were visualized and measured using the 3D software. The process of intersecting the proximal and distal osteotomized segments to measure the interference is called “Boolean operation.” The Boolean process was performed separately for both sides, and the 3D visualization of the interference was displayed. Information on the volume (mm^3^) and dimensions of the interference is automatically tabulated in the 3D properties of the resultant interference (Fig. 1B).

### Surgical procedures

**Fig. 2:**
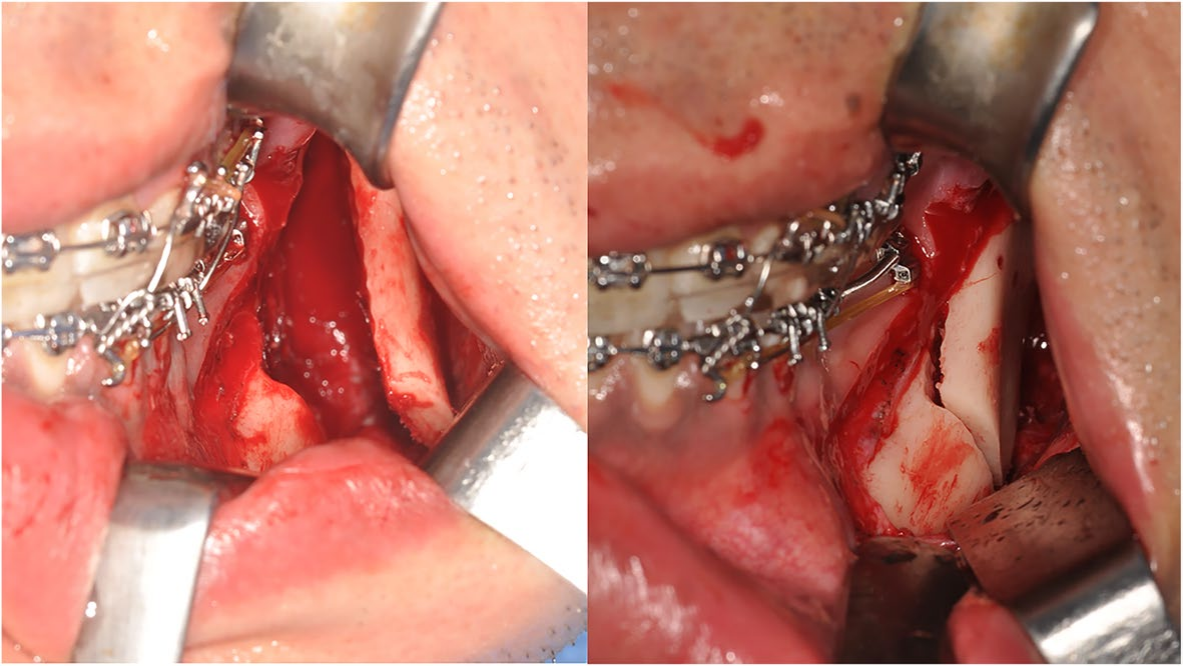
The 3D virtual prediction process was considered intraoperatively. The predicted interosseous interference presented in Fig. 1 was also noted during the surgery. The premature contacts between the proximal and distal segments (left) were reduced by grinding the medial surface of the proximal segment (right)

For SSRO, the osteotomy cut starts from the lingual surface of the ascending ramus just above the lingula to the posterior border of the mandibular ascending ramus horizontally extending to the anterior ramus and the superior surface of the body of the mandible. Vertical osteotomy is performed on the body of the mandible over the second molar area including the inferior border. After the osteotomy cuts, a thin osteotome is malleted into the osteotomy sites, and the procedure is repeated on the opposite side. In this study, any bony interference that had been shown in the 3D virtual prediction process was considered intraoperatively. During the operation, the potential interference between the bone segments was thoroughly checked again. These premature contacts were reduced by various additional surgical procedure of original Obwegeser osteotomy including the vertical cutting of the posterior part of the distal segment, grinding of the medial surface of the proximal segment, or addition of lingual osteotomy [7, 13–15]. These procedures ensure adequate proximal segment positioning and passive condyle seating without condyle displacement. In addition, the passive adaptation of the proximal segment increases the interosseous contact surface with the distal segment (Fig. 2).

### Outcome assessments

**Fig. 3:**
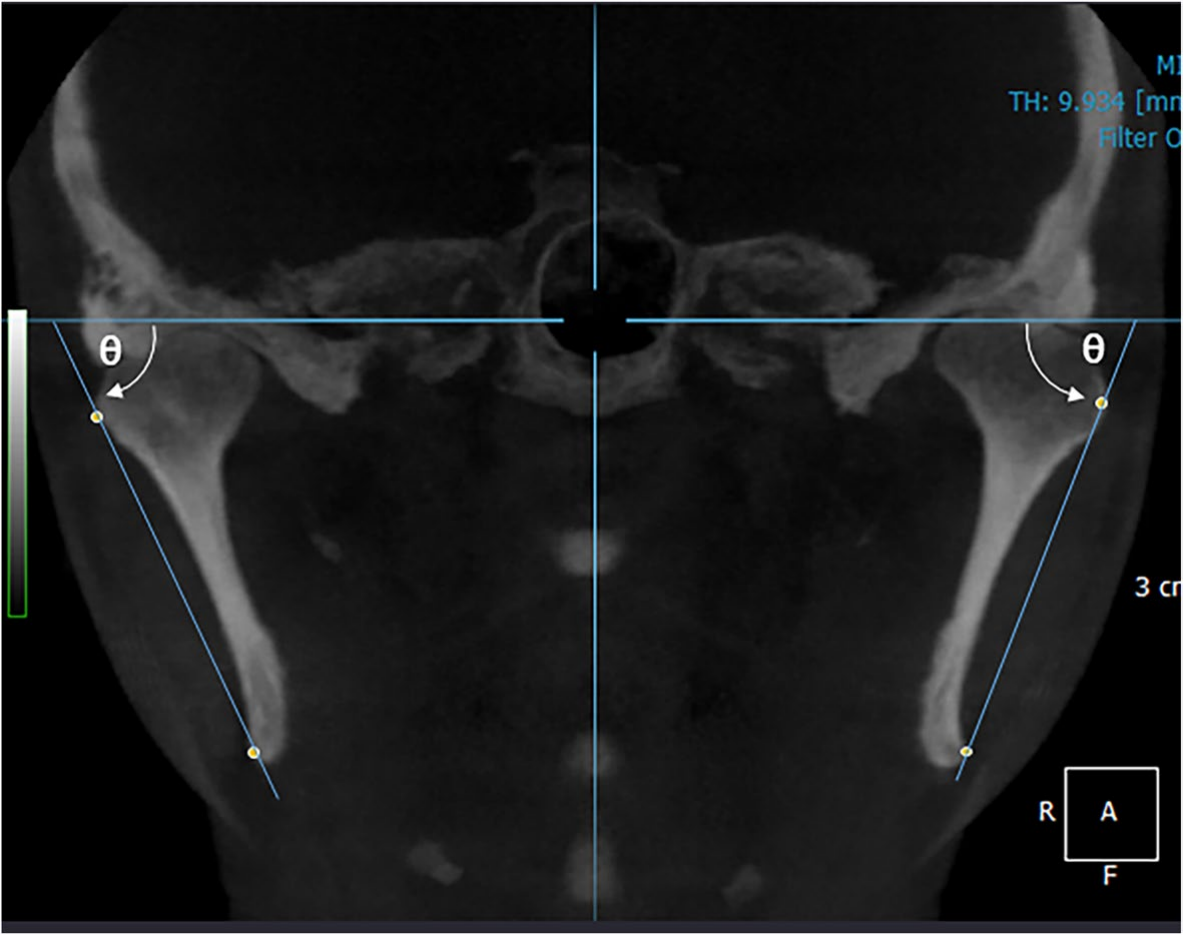
The ramal inclination, the angle between the line between the superolateral point on the condyle, and the gonion relative to the FH plane

The 3D coordinate system was constructed using anatomical reference points and reference planes as previously described [16]. Briefly, the Frankfort horizontal plane was defined as the horizontal reference plane, and the sagittal reference plane was the plane perpendicular to the FH plane and passing through the nasion and basion. Anteroposterior position and transverse movements were evaluated using pogonion (Pog) and Me, respectively. Ramal inclination was defined as the angle between the line from a superio-lateral point on the condyle (C) and the most inferior and posterior point on the mandibular gonion angle (gonion, G) to FH plane (Fig. 3). The process of “mirroring” was performed, where the “short side” was represented as the side in which the mandibular midline had shifted from the midfacial reference plane (deviated side) and the “long side” was interpreted as the contralateral side to the mandibular deviation. The Dahlberg method error for 3D measurement was 0.45–1.32 mm for linear measurements and 0.53–0.95° for angular measurements, which was not statistically significant.

### Data analysis and statistics

Data were statistically analyzed to record the postoperative changes and relapse. The paired *t*-test was employed to analyze the bilateral difference or T1–T0 and T2–T1 differences in the three groups. Intergroup differences were compared with the one-way ANOVA followed by Tukey’s post hoc test. The correlation between Me deviation, the volume of interosseous interference, and bilateral ramal inclination was analyzed using Pearson’s correlation analysis. The potential influence of the age, gender, amount of mandibular setback, and ramal inclination on each side on the predicted volume of interosseous interference of both sides was investigated using stepwise linear regression analysis. Statistical significance was set at a *p*-value of < 0.05. Statistical analysis of the measurements was conducted using SPSS version 12.0 (SPSS Inc., Chicago, IL, USA).

## Results

### Demographic features of the groups

This study recruited 116 patients (male:female = 67:49). The age of the patients ranged from 16 to 43 years (the mean age of 22.1 ± 3.7) at the time of orthognathic surgery. Group 1 (*n* = 33, male: female = 20:13) had a mean age of 21.7 ± 3.5 years and Me deviation of 0.4 ± 0.7 mm, Group 2 (*n* = 39, male:female = 17:22) had a mean age of 21.8 ± 4.1 years and Me deviation of 2.8 ± 0.6 mm, and Group 3 (*n* = 44, male:female = 30:14) had a mean age of 22.6 ± 3.5 years and Me deviation of 5.9 ± 1.7 mm (Table 1).

**Table 1:** Demographic characteristics of the patients according to the groups

Characteristics	Group 1	Group 2	Group 3
Sample size (*n*)	33	39	44
Sex, male *(*%)	20 (60.6%)	17 (43.6%)	30 (68.2%)
Age, years, mean ± SD (range)	21.7 ± 3.5 (17 ~ 34)	21.8 ± 4.1 (18 ~ 35)	22.6 ± 3.5 (17 ~ 36)
Me deviation, mm, mean ± SD (range)*	0.4 ± 0.7 (0 ~ 1.9)	2.8 ± 0.6 (2.0 ~ 3.9)	5.9 ± 1.7 (4.0 ~ 12.0)

*SD* standard deviation. Sex and age distribution were not significantly different among the groups

^*^Significant intergroup difference was noted among the groups in Me deviation (ANOVA, *p* < 0.001)

### Interosseous interference between the proximal and distal segments

**Fig. 4:**
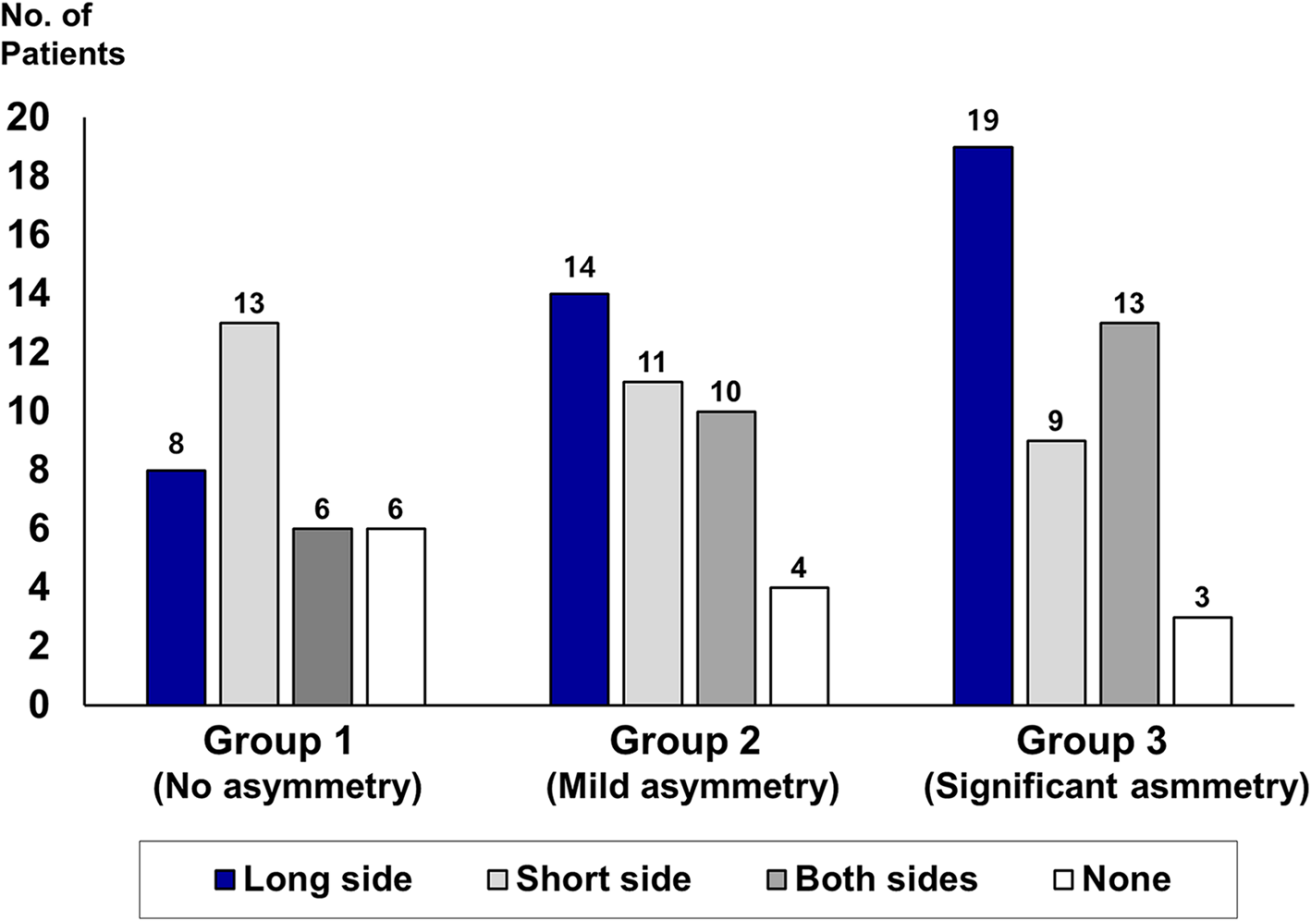
Anticipated site of interosseous interference between the proximal and distal segments in computer-assisted surgical simulation according to the groups

The pattern of 3D interosseous interference between the proximal and distal segments has been analyzed in the three groups. In Group 1, osseous interference was noted unilaterally (*n* = 21, 63.6%) or bilaterally (*n* = 6, 18.2%). Six patients did not show the site of bony collision on both sides (*n* = 6). In Groups 2 and 3, unilateral interosseous interference was dominant [*n* = 25 (64.1%), *n* = 28 (63.7%), respectively] over bilateral interference (25.6%, 29.5%, respectively). In asymmetry patients, the anticipated interosseous interference was more frequently noted on the long side (35.9% in Group 2, 43.2% in Group 3) rather than the short side (28.2% in Group 2, 20.5% in Group 3) (Fig. 4).

Table 2 represents the predicted volume of interosseous interference according to the groups. No significant bilateral difference in the volume of interosseous interference was noted in Groups 1 and 2, whereas a significantly greater interosseous interference was observed on long side (177.7 ± 348.4 mm^3^) compared to the short side (54.4 ± 124.4 mm^3^) in Group 3 (*p* = 0.033).

**Table 2:** Three-dimensional volume of interosseous interference in the osteotomized mandibular segments in computer-assisted surgical simulation (mm^3^)

	**Group 1** **Mean ± SD (range)**	**Group 2** **Mean ± SD (range)**	**Group 3** **Mean ± SD (range)**
Long side	30.2 ± 77.2 (0 ~ 389.3)	58.5 ± 158.8 (0 ~ 843.8)	186.0 ± 343.0 (0 ~ 1445.5)
Short side	39.6 ± 89.8 (0 ~ 445.1)	22.0 ± 42.2 (0 ~ 176.2)	54.4 ± 124.4 (0 ~ 586.9)
ΔLong-short side	− 9.4 ± 127.7 (− 445.1 ~ 389.3)	36.6 ± 171 (− 176.2 ~ 843.8)	122.3 ± 395.4 (− 586.9 ~ 1445.5)
*p*	0.676	0.190	0.033

*SD* standard deviation. *p*-value, bilateral difference from paired t

### Changes in mandibular ramal inclination and chin points

**Fig. 5:**
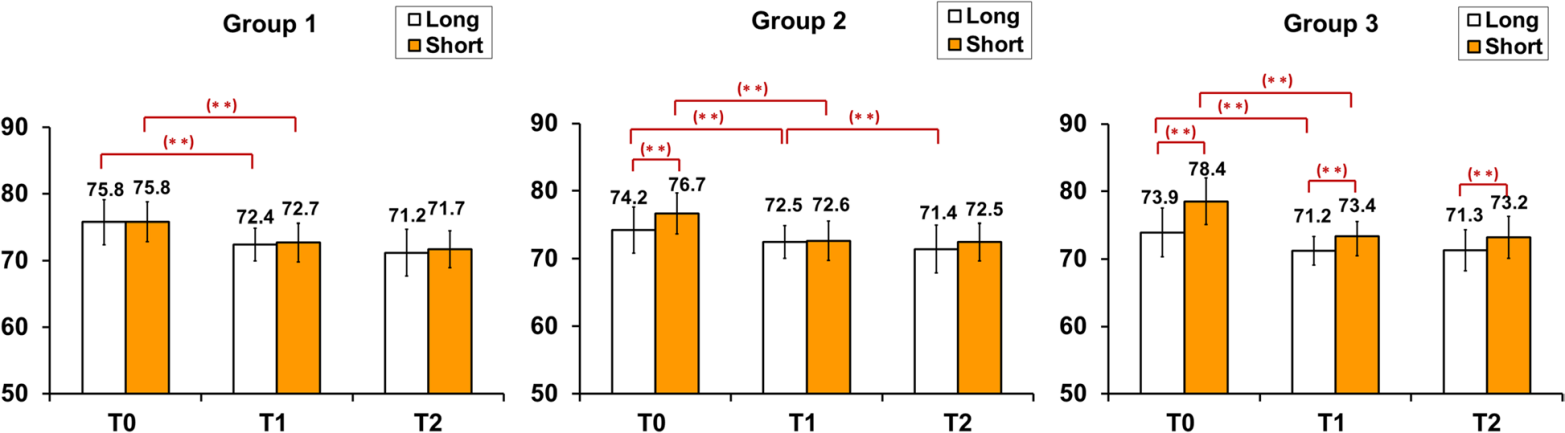
Preoperative (T0), immediate postoperative (T1), and at least 6-month postoperative (T2) changes in bilateral ramal inclination. Differences between the sides or examination period were evaluated using paired *t*-test (**p < 0.01)

In Group 1, there was no bilateral difference at T0, T1, and T2. Bilateral ramal inclination narrowed after surgery but did not exhibit statistical difference. Groups 2 and 3 showed significant bilateral differences in ramal inclination before surgery. When surgical changes were compared (ΔT1–T0), all three groups exhibited a significant reduction in ramal inclination in both the long and short sides after surgery (all *p* < 0.001). These reductions remained stable postoperatively (ΔT2–T1, all *p* > 0.05) in Groups 1 and 3 (Fig. 5).

The transverse position of the chin (Me) showed significant changes after surgery (ΔT1–T0; Group 1, − 0.3 ± 0.6 mm, *p* = 0.022; Group 2, − 2.4 ± 1.1 mm, *p* < 0.001; Group 3, − 5.6 ± 1.7 mm,* p* < 0.001). In all three groups, the transverse Me position remained stable postoperatively (ΔT2 − T1, all *p* > 0.05). On the other hand, the anteroposterior position of the chin (Pog) showed significant relapse postoperatively (ΔT2 − T1; Group 1, 0.8 ± 0.8 mm; Group 2, 0.7 ± 0.7 mm; Group 3, 1.0 ± 1.7 mm; all* p* < 0.001) (Table 3).

**Table 3:** Difference in transverse position of Me and anteroposterior position of Pog at T0, T1, and T2 in the 3 groups

	**Group 1**		**Group 2**		**Group 3**	
	Mean ± SD	*p*	Mean ± SD	*p*	Mean ± SD	*p*
Me (transverse)						
∆T1–T0	− 0.3 ± 0.6	0.022	− 2.4 ± 1.1	< 0.001	− 5.6 ± 1.7	< 0.001
∆T2–T1	0.0 ± 0.2	0.402	0.1 ± 0.6	0.498	0.0 ± 0.2	0.427
Pog (antero-posterior)						
∆T1–T0	− 9.2 ± 6.2	< 0.001	− 8.4 ± 5.7	< 0.001	− 7.8 ± 6.3	< 0.001
∆T2–T1	0.8 ± 0.8	< 0.001	0.7 ± 0.7	< 0.001	1.0 ± 0.5	< 0.001

Negative value indicates transverse movement of Me to contralateral side of chin deviation and posterior movement at Pog

**Fig. 6:**
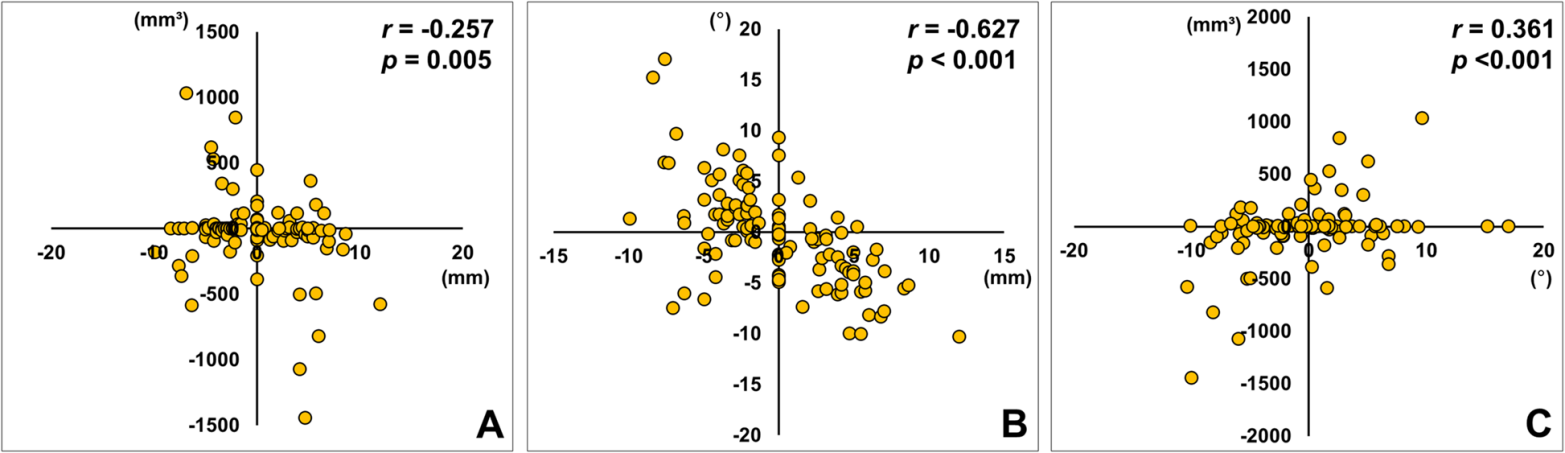
**A** Correlation between the Me deviation (mm, positive value indicate left side) before surgery (T0) and predicted bilateral difference in the volume of interosseous interference (ΔLt-Rt, mm^3^). **B** Correlation between the Me deviation and bilateral difference in ramal inclination (ΔLt-Rt, °). **C** Correlation between the predicted bilateral difference in the volume of interosseous interference and bilateral difference in ramal inclination

The correlation analysis revealed that the amount of Me deviation (T0) was significantly correlated with the bilateral difference in the volume of interosseous interference (ΔLt-Rt) predicted by the CASS (*r* = −0.257, *p* = 0.005) (Fig. 6A). Furthermore, the Me deviation (T0) was significantly correlated with the bilateral difference in ramal inclination (*r* = −0.627, *p* < 0.001) (Fig. 6B). The side with more mandibular setback (long side) exhibited narrower ramal inclination and more 3D interosseous interference. The bilateral difference in ramal inclination was significantly correlated with interbony interference (*r* = 0.361, *p* < 0.001) (Fig. 6C).

To determine the important predictor variables affecting the volume of interosseous interference, stepwise regression analysis was performed. Demographic variables (age, gender), nominal variable (long vs short side), or amount of mandibular setback at Pog did not influence the volume of interference. Degree of chin asymmetry (amount of Me deviation) and the degree of ramal inclination significantly influenced the predicted 3D volume of bony collision in the statistical model. The major predictor variable that affected the volume of bony interference was the amount of Me deviation (*p* = 0.010) (Table 4).

**Table 4:** Stepwise regression analysis for parameter estimates of independent variables affecting predicted volume of interosseous interference

Variables	Unstandardized coefficients		Standardized coefficients	95% Cl for B (lower ~ upper bound)	*P*
	*B*	Standard error	Beta		
Model 1 (constant)	28.052	19.903		− 11.164 ~ 67.269	0.160
Me deviation	12.395	4.760	0.169	3.017 ~ 21.774	0.010
Model 2 (constant)	724.183	252.280		227.097 ~ 1221.269	0.004
Me deviation	13.184	4.701	0.180	3.922 ~ 22.446	0.005
Ramal inclination	− 9.215	3.330	− 0.178	− 15.776 ~ − 2.655	0.006

Excluded variables: gender, age, side (long vs short), and amount of mandibular setback. 95% Cl, 95% confidence interval

## Discussion

In this study, we analyzed the predicted volume of interosseous interference between the proximal and distal segments of the osteotomized mandible before surgery. Using the 3D information of the predicted bony interference by occlusion-based surgical simulation, the bony interference could be clearly defined. The purpose of this study was to address the following questions: (1) which side shows more interosseous interference? and (2) what is the major influencing factor for interosseous interference in SSRO for asymmetric mandibular prognathism? To answer the key questions, we investigated (1) the pattern and volume of interosseous interference according to the groups and (2) the predictor variable that affected the volume of osseous interference.

The results of the current study showed the following: (1) the anticipated interosseous interference was more frequently noted on the contralateral side of deviation (long side) than the deviated site (short side). However, osseous interference could be existed on any side, unilaterally or bilaterally. (2) Amount of Me deviation and the degree of ramal inclination significantly influenced the predicted 3D volume of bony collision. Age, sex, amount of setback at Pog, or side (long vs short) was excluded from influencing factor to interference volume.

The interbony interference can directly cause changes in the condylar position and influence the temporomandibular function and long-term stability. Bony interference can occur not only in the horizontal but also in the vertical plane, particularly when the mandibular distal segment is mobilized with yaw, roll, or pitch rotation. In general, the more the chin deviation, the greater the amount of bone interference. Our result indicated that the amount of interference was larger on the long side (177.7 ± 348.4 mm^3^) than on the short side (54.4 ± 124.4 mm^3^). Furthermore, the contralateral side of deviation (long side) showed a narrower ramal inclination than the deviated side (short side), and the amount of asymmetry was proportional to the difference in the volume of bone interference. Simultaneously, the bilateral differences in ramal inclination were significantly correlated with the volume of bone interference. The results suggest that on the side with more mandibular setbacks (long side), more interferences can be anticipated and need to be considered before surgery. We investigated the side with greater interference volume. In the case of severe asymmetry (group 3), there was more interference on the long side (*n* = 29) than on the short side (*n* = 21). These findings indicated that even though it can be anticipated that there would be more interference on the long side, the short side also can have interosseous interferences. This means that osseous interference could be existed on any side, unilaterally or bilaterally in patients with or without asymmetry.

**Fig. 7:**
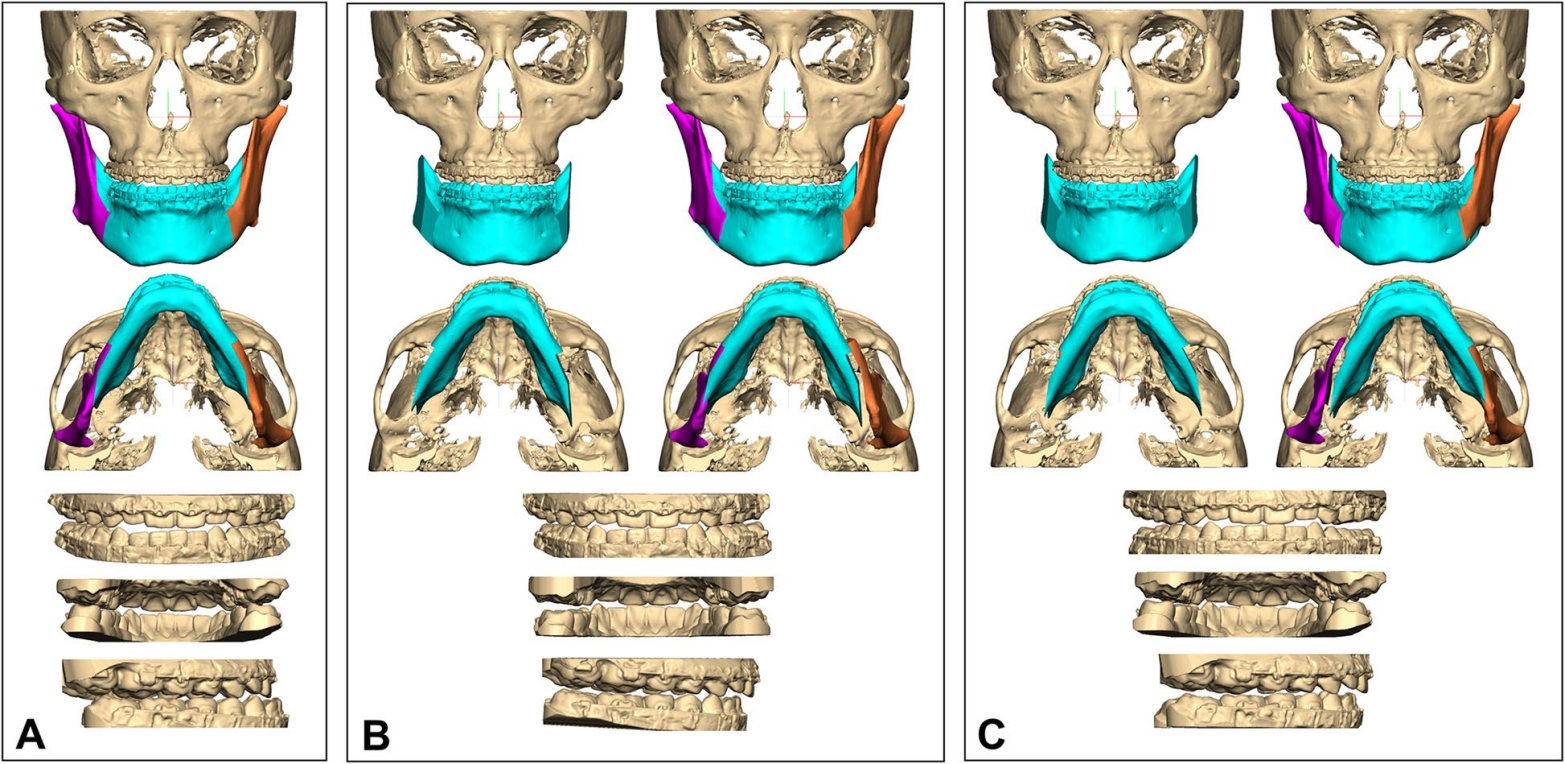
Different setup occlusion can result in different sites of interference or gap. The severity or location of interosseous interference is determined by the transverse or rotational movement of the mandibular distal segment according to the planned occlusion and skeletal position. **A** Preoperative occlusion and skeletal position in a patient with mandibular deviation to the right side. **B** Simulation #1, 3D simulation based on a planned occlusion resulted in interosseous interference on the right side and a gap on the left side. **C** Simulation #2, another set-up occlusion shows interosseous interference on the left side and a gap on the right side

We usually perform CASS based on the simulated postoperative occlusion and intercuspation; distal mandibular segments would be mobilized in various directions. Only the 3D-simulated image can be seen after finishing the occlusion-based segment mobilization, which is a complex spatial movement. Therefore, it cannot be the same as a 2-dimensional prediction from posteroanterior cephalometry. Different types of set-up occlusion can result in different sites of bony interference or gap (Fig. 7). The site and volume of interosseous interference could be determined by the complex direction and amount of yaw or roll rotation of the distal segment according to the planned occlusion and skeletal position change. Therefore, preoperative 3D surgical simulation is important to minimize osseous interference and successfully control the bony collision. Our results indicated that 3D interference needs to be individually analyzed and surgical modification be the patient-specific condition.

Various efforts have been made to reduce the bony interference between the proximal and distal bone segments. SSRO modification was suggested by changing the cutting design [13, 17–19], resection of interfering bony part (or mandibular angle) in proximal segments [20], or intentional posterior ostectomy of the distal segment had been added after the sagittal splitting [13, 21]. When severe interference was anticipated, secondary lingual osteotomy was recommended [5, 15, 22]. In a previous study, maxillary surgery was added to mobilize the mandibular distal segment-maxilla complex simultaneously to minimize the mandibular proximal–distal segment interference [6].

If we can establish a threshold (cutoff value) for the level of interference, it would be helpful to decide to add a specific surgical approach if the projected volumetric interference surpasses a specific threshold. However, it was difficult to establish such a cutoff value or threshold to select the specific surgical technique because the volume of interference was not directly correlated with the real severity of interference at the operation theater. For example, even though there were similar degrees of asymmetry, some patient shows a large volume of interference at the anterior-lingual region of proximal segments that can be removed by simple grinding. However, another patient shows a narrow but deep bony collision at the posterior part of the distal segment, which can be managed by lingual osteotomy.

In our institution, the aforementioned techniques are currently used in the operation room to eliminate the premature contact of the segments that interferes with passive segment adaptation. If the volume of interference is not significant, grinding the lingual side of the proximal segment is usually enough. However, if severe interference is present, posterior ostectomy or lingual osteotomy is accompanied during the SSRO surgery. Previous reports also showed that the prediction of premature bony contact or interosseous interference can be efficiently simulated from the CASS and is beneficial for surgeons [5, 6]. Ramal inclination can be intentionally changed for esthetic purposes in our clinic. For example, ramal inclination at the short side is wider than the long side before surgery. Furthermore, ramal inclination on the short side needs to be adjusted and narrowed to match similar to the long side. Therefore, intentional change in ramal inclination change is necessary for asymmetry patients. Oriental patients usually want a slender facial shape rather than a square shape. Hence, they prefer narrowing the ramal inclination rather than maintaining the original ramal inclination after SSRO. For functional and esthetic purposes, the elimination of osseous interference and ramal inclination change would increase patient satisfaction.

Our results indicated that the transverse Me position was stable after significant narrowing of the bilateral ramal angle after the surgery. Not only the symmetry group but also the asymmetry group showed a significant reduction in ramal inclination, and the ramal inclination was maintained during the postoperative follow-up period (ΔT2–T1). Although there were significant anteroposterior relapses, the amount was nearly 1 mm, which is not greater than in previous reports [23, 24].

Using CASS, accurate diagnosis can be obtained for the surgery which can improve patient safety and the outcomes [10]. The 3D simulation also facilitates the virtual splitting of the skull into separate segments and 3D movements of bone segments to determine the final position of the separated mandibular segment [25].

To the best of our knowledge, this is the first study to quantify the volume of interference and investigated the influencing factor to osseous interference. We could collect data on interosseous interference through subtraction of proximal and distal osteotomized segments using Boolean operation in the computer software. This method would also be useful for analyzing the anticipated segment collision in any type of orthognathic surgery.

This study and other previous studies had limitations that need to be acknowledged. In general, the actual osteotomy in the operation room is not the same as the 3D virtual osteotomy in the CASS. Thus, although it does not influence the outcomes, the osteotomized segment would be different from virtual planning. Valls-Ontanon (2020) reported that 3D CASS was sensitive (100%) but showed low specificity (51.6%) in predicting proximal and distal segment interferences [5]. Further development of CASS needs to proceed to implement more precise and predictable 3D virtual planning. In this study, we did not experience TMJ disorder or significant functional problems after changing the ramal inclination in our patients. However, a detailed clinical examination was not conducted in this study. Further study is needed to clarify this issue.

## Conclusion

Computer-assisted simulation can predict interosseous interference or gap after orthognathic surgery for patients with facial asymmetry. The major determinant for the volume of the osseous interference between the proximal and distal segments was the severity of chin deviation. The osseous interference can be existed on any side, unilaterally or bilaterally. This is because the severity or location of interosseous interference could be determined by the transverse or rotational movement of the mandibular distal segment according to the planned occlusion and skeletal position. 3D surgical simulation is necessary before surgery to predict osseous interference and improve the ramal inclination.

## Data Availability

The datasets used and/or analyzed during the current study are available from the corresponding author on reasonable request.

## Abbreviations

SSRO: Sagittal split ramus osteotomy
CBCT: Cone-beam computed tomograph
TMJ: Temporomandibular joint
3D: Three dimensional
CASS: Computer-assisted simulation system
Pog: Pogonion
